# Suppression of Calcium Entry Modulates the Expression of TRβ1 and Runx2 in Thyroid Cancer Cells, Two Transcription Factors That Regulate Invasion, Proliferation and Thyroid-Specific Protein Levels

**DOI:** 10.3390/cancers14235838

**Published:** 2022-11-26

**Authors:** Muhammad Yasir Asghar, Taru Knuutinen, Emilia Holm, Tommy Nordström, Van Dien Nguyen, You Zhou, Kid Törnquist

**Affiliations:** 1 Cell and Tissue Dynamics Research Program, Institute of Biotechnology, HiLIFE, University of Helsinki, Viikinkaari 9, FI-00014 Helsinki, Finland; 2 Minerva Foundation Institute for Medical Research, Biomedicum, Helsinki 2U, Tukholmankatu 8, FI-00290 Helsinki, Finland; 3 Faculty of Science and Engineering, Cell Biology, Åbo Akademi University, Tykistökatu 6A, FI-20520 Turku, Finland; 4 Department of Physiology, Faculty of Medicine, Biomedicum Helsinki, University of Helsinki, FI-00014 Helsinki, Finland; 5 Division of Infection and Immunity, School of Medicine, Systems Immunity University Research Institute, Cardiff University, Cardiff CF10 3AT, UK

**Keywords:** thyroid hormone receptor beta 1 (TRβ1), Runt-related transcription factor 2 (Runx2), invasion and migration, thyroid cancer, metastasis, proliferation, calcium

## Abstract

**Simple Summary:**

Thyroid cancer is the most common endocrine cancer and the treatment for such hostile tumors remains a complex challenge. Thus, new insights and mechanisms need to be explored, to design a most suitable therapy for thyroid cancer patients. In this study, we found that the expression of thyroid hormone receptor beta 1 (TRβ1) and Runt-related transcription factor 2 (Runx2) is calcium dependent. The expression of TRβ1 was downregulated but Runx2 upregulated in all investigated thyroid cancer cell lines compared to normal primary thyroid cells. The restoration of TRβ1 expression in thyroid cancer cells inhibited proliferation, invasion and restored thyroid specific proteins expression. Conversely, inhibiting Runx2 decreased proliferation and invasion but had no effect on expression of thyroid specific proteins. We present a novel strategy where inhibiting calcium entry, restoration of TRβ1 and blocking Runx2 can serve as potential therapeutic targets.

**Abstract:**

The thyroid hormone receptor beta 1 (TRβ1) is downregulated in several human cancer cell types, which has been associated with development of an aggressive tumor phenotype and the upregulation of Runt-related transcription factor 2 (Runx2). In this study, we show that the expression of TRβ1 protein is downregulated in human thyroid cancer tissues and cell lines compared with the normal thyroid tissues and primary cell line, whilst Runx2 is upregulated under the same conditions. In contrast, the expression of TRβ1 is upregulated, whereas Runx2 is downregulated, in STIM1, Orai1 and TRPC1 knockdown cells, compared to mock transfected cells. To study the functional significance of Runx2 in follicular thyroid cancer ML-1 cells, we downregulated it by siRNA. This increased store-operated calcium entry (SOCE), but decreased cell proliferation and invasion. Moreover, restoring TRβ1 expression in ML-1 cells decreased SOCE, basal and sphingosine 1-phosphate (S1P)-evoked invasion, the expression of the promigratory S1P3 receptor and pERK1/2, and at the same time increased the expression of the thyroid specific proteins thyroglobulin, thyroperoxidase, and thyroid transcription factor-1. In conclusion, we show that TRβ1 is downregulated in thyroid cancer cells and that restoration of its expression can reverse the cancer cell phenotype towards a normal thyroid cell phenotype.

## 1. Introduction

Among the endocrine forms of cancer, thyroid cancer is the most common. In Finland, the incidence of thyroid cancer has increased in both men and women during the past decades. However, the mortality has declined in women since the early 1990s. The survival ratio is currently around 95% for women and around 85% for men [1] (https://syoparekisteri.fi/assets/files/2022/06/Cancer-2020-report_eng.pdf; accessed on 4 October 2022). Furthermore, according to the American Cancer statistics 2022 report, the incidence rate of thyroid cancer has declined by 2.5% per year during 2014–2018 after decades of a steady increase. The estimated number of new thyroid cancer cases for the year 2022 is 43,800 with an estimated death rate of 2230 individuals. The probability of developing invasive thyroid cancer during a person’s lifetime is 0.7% [2]. The 5-years survival rate for papillary and follicular thyroid cancer is 95% and 91% respectively. However, it is only 1–7% for anaplastic thyroid cancer patients.

Metastatic thyroid cancer is among the most lethal endocrine malignancies, and the patients have a minimal survival rate of 3 to 5 months [3]. A rational treatment of such an invasive phenotype of thyroid cancer remains a complex challenge that requires further insights and novel approaches [4,5].

The thyroid hormone receptors (TRs) are nuclear transcription factors encoded by two genes, TRα and TRβ, and the latter has two isoforms TRβ1, and TRβ2. These receptors modulate a multitude of cellular processes, including cell metabolism, differentiation, growth, and development [6,7,8,9,10,11]. Somatic mutations in TRα and TRβ genes have been reported in different cancers including, hepatocarcinoma, pituitary tumors, renal carcinoma and thyroid cancer. Most of these mutations impair the binding of triiodothyronine (T3) to its receptors, or the DNA binding of TRs, leading to an aggressive phenotype [12]. TRβ1 has been shown to function as a tumor suppressor in several cancer cells, including thyroid cancer cells. Thus, overexpression of TRβ1 decreased tumor growth, invasion, and metastasis in thyroid and breast cancer cells [11,13,14].

Runt-related transcription factors (Runx) are a family of nuclear heterodimeric transcription factors, consisting of three factors entitled Runx1, Runx2 and Runx3. These factors are important in e.g., the formation of bone and in neural development but have also been implicated in the progression of cancer [15,16,17]. Among these factors, Runx2 is important for the bone formation and mineralization, and may also regulate proliferation and differentiation of chondrocytes [17,18,19,20]. Moreover, Runx2 has also been shown to have oncogenic properties, and an up-regulation of Runx2 has been correlated with a worsened prognosis of several cancers, including thyroid cancer. The effect of Runx2 is thought to be mediated by the PI3K/Akt pathway, and by an up-regulating of the anti-apoptotic factor Bcl-2 [17,19,20,21].

We have previously shown that attenuation of calcium entry potently decreased thyroid cancer cell proliferation, as well as invasion and migration, by decreasing e.g., ERK expression and phosphorylation, and by attenuating matrix-metalloproteinase expression and secretion. In addition, attenuating calcium entry increased the expression of thyroid specific proteins [22,23]. In the present work, we investigated whether inhibiting calcium entry may modulate the expression of TRβ1 or Runx2, two important transcription factors in the thyroid. To the best of our knowledge, no information is available regarding whether these two transcription factors are regulated by calcium signaling in thyroid cells. Furthermore, we wanted to investigate whether modulating TRβ1or Runx2 expression affected the phenotype of the thyroid cancer cells. Our results show that attenuating calcium entry in the cells increased the expression of TRβ1, whereas the expression of Runx2 was decreased. Furthermore, overexpressing TRβ1 increased the levels of thyroid-specific proteins, but decreased proliferation and invasion of the cells. Downregulating Runx2 decreased both proliferation and invasion of the cells. Thus, both TRβ1 and Runx2 are regulated by calcium signals, and by overexpressing TRβ1 the cancer cells obtained a more normal thyroid cell phenotype.

## 2. Materials and Methods

### 2.1. Materials

Human primary thyroid epithelial cells (Cell Biologics, Chicago, IL, USA). FTC-133 follicular thyroid cancer cells (Banca Biologica e Cell Factory, Genova, Italy). C643 anaplastic thyroid cancer cells (Dr Nils-Erik Heldin, Karolinska Institute, Stockholm, Sweden). The THJ-16T anaplastic thyroid cancer cells (Dr John Copland, Mayo Clinic, Jacksonville, FL, USA). DMEM, shRNA lentivirus particles, poly-L-lysine, BSA, fatty acid-free BSA, puromycin, HEPES, sodium pyruvate (Sigma-Aldrich, St. Louis, MO, USA), RPMI 1640 (Lonza, Basel, Switzerland), F-12 Ham’s GIBCO, H6621 with supplements, gelatin coating (Cell Biologics, Chicago, IL, USA), FBS, penicillin/streptomycin, L-glutamine, trypsin–EDTA, bicinchoninic acid protein assay BCA kit, OptiMEM (Thermo Fisher Scientific, Waltham, MA, USA), Fura-2 AM (Molecular Probes, Eugene, OR, USA), Thapsigargin (Tg) (Alexis Corporation, San Diego, CA, USA), Sphingosine 1-phosphate (S1P) (Biomol International, Plymouth Meeting, PA, USA), Hsc70 antibody (Enzo Life Sciences, Inc., Farmingdale, NY, USA), TRβ1 (J51, J52), S1P_3_, S1P_1_, TPO, TG, NIS, TTF-1 antibodies (Santa Cruz Biotechnology, Santa Cruz, CA, USA). Runx2 antibody, HRP-conjugated goat anti-rabbit IgG (Abcam, Waltham, MA, USA). p21waf1/cip1, p27kip1, ERK1/2, pERK1/2, β-actin, HRP-conjugated anti-rat and anti-mouse IgG antibodies (Cell Signaling Technology, Danvers, MA, USA). HRP-conjugated donkey anti-goat antibody (Promega, WI, USA). Cell culture plastic ware, human collagen type IV (Becton Dickinson, NJ, USA), Transwell inserts (Corning, NY, USA), Fura-2 AM (Molecular probes, Eugene, OR, USA), Thapsigargin (Alexis Co., San Diego, CA, USA). All chemicals used in this study were molecular biology grade.

### 2.2. Cell Culture

Human primary thyroid epithelial cells (P.T) were cultured in H6621 medium with supplements according to the manufacturer’s instructions. ML-1 cells were cultured in DMEM with 10% FBS, 1% penicillin/streptomycin (P/S), and 1% L-glutamine. FTC-133 cells were cultured in DMEM and F-12 (Ham’s) medium (1:1) with 10% FBS and 1% L-glutamine and 1% P/S. C643 cells were cultured in DMEM with 10% FBS, 1% L-glutamine, and 1% penicillin/streptomycin. THJ-16T cells were cultured in RPMI 1640 with 10% FBS, 1% penicillin/streptomycin, 1 mM sodium pyruvate, and 25.03 mM HEPES. All cell cultures were maintained in a water-saturated atmosphere with 5% CO2 and 95% air at 37 °C in the incubators.

### 2.3. Lentiviral Transduction and Generation of Stable Cell Lines

The cell lines used have been described before [22,23]. In short, human follicular thyroid cancer ML-1 cells were seeded on 12-well plates and grown for up to 70% confluency followed by the lentiviral transduction using non-targeting shRNA, and STIM1-, ORAI1-, or TRPC1-targeting lentiviral particles according to the lentiviral transduction protocol (Sigma, St. Louis, MO, USA). The short hairpin (shRNA) sequences are presented in Table 1. After 48 h of post transduction, the cells were cultured and maintained in DMEM medium containing 0.5 µg/mL Puromycin.

### 2.4. Transient Transfections

The pellets of 4 million cells were re-suspended in 400 µL OptiMEM together with plasmids (20 µg of pcDNA or pTRβ1) or siRNA (2 µM of the control siRNA or siRUNX2). The cells were electroporated at 975 µF and 240 V and grown in respective cell culture medium for 48 h (with a change of media to fresh medium after 24 h) before the start of each experiment. The sequences of plasmids and siRNAs are presented in Table 1. The control plasmid (pcDNA3) was purchased from Addgene MA, USA. The pTRβ1 was shared by Dr. Sheue-Yann Cheng (National Institute of Health, Bethesda, MD, USA). The siTRβ1, siRunx2 and the non-targeting control siRNA were designed and purchased from Eurofins Genomics (Ebersberg, Germany).

### 2.5. Western Blot Analysis

The whole cell lysates were made with lysis buffer (10 mM Tris-HCl, 150 mM NaCl, 7 mM EDTA and 0.5% NP-40) on ice and protein concentrations were measured with the BCA protein assay kit. Then, the Laemmli sample buffer (LSB) was added to each lysate sample. Equal amounts of samples were loaded and the proteins were separated by SDS-PAGE. Next, the proteins were blotted on nitrocellulose membrane [24]. In some experiments, the proteins were blotted onto PVDF membranes using BioRad Transblot system. After blocking in 5% fat-free milk in TBS, the blots were incubated for overnight at +4 °C with respective primary antibodies TRβ1 J51 (1:200) or J52 (1:100), Runx2 (1:500), S1P1 (1:500), S1P3 (1:500), pERK1/2 (1:500), ERK1/2 (1:500), p21 (1:1000), p27 (1:1000), TPO (1:200), TTF-1 (1:200), TG (1:200), NIS (1:200) and Hsc70 (1:4000). Next day, the blots were incubated in respective secondary antibody as HRP-conjugated goat anti-rabbit (1:2000), HRP-conjugated anti-mouse (1:3000), HRP-conjugated anti rat (1:3000) and HRP-conjugated donkey anti-rabbit (1:10,000).

Proteins bands were detected with enhanced chemiluminescence (ECL; Thermo Scientific, Waltham, MA, USA). Densitometric analysis was done by using Fiji-ImageJ software. The intensities of each protein were normalized to either Hsc70 or β-Actin expression, respectively. The results are presented as expression of protein (%).

### 2.6. Proliferation Assays

The proliferation of the ML-1 cells was investigated using [^3^H] thymidine incorporation assay method [23]. Briefly, 100,000 (48 h, post-transfection) cells were grown on 35 mm plates for 20 h. Then, 0.4 μCi/mL [^3^H] thymidine was added to each plate for 4 h. The radioactivity was measured as counts per minute (cpm) using a Wallac 1414 liquid scintillation counter. The results were normalized and presented as proliferation (%).

### 2.7. FACS Analysis

0.5 million cells were seeded on 35-mm plates and the cells were allowed to grow overnight. Next day, the cells were detached and centrifuged to form pellets. 500 μL of propidium iodide solution containing 0.05 mg/mL propidium iodide, 3.8 μM sodium citrate, and 0.1% Triton X-100 in PBS was added to each sample pellet, mixed and incubated for 15 min at room temperature in dark. The samples were then processed by flow cytometry using Novocyte Quanteon 4025 system (Agilent, Santa Clara, CA, USA) followed by analysis using the Novoexpress flow cytometry software in Cell Cycle analysis module to calculate the percentage of cells in each phase of cell cycle.

### 2.8. Migration and Invasion Assays

The invasion assays were performed by using Transwell inserts method as described previously [23]. Briefly, 50,000 cells in serum free medium (No FBS) were allowed to migrate towards 10% FBS containing medium for 16 h. In experiments to study the effect of bioactive lipid S1P, the cells were lipid starved by changing the medium to 0.2% FAF-BSA-containing serum-free medium (SFM) for overnight. Next day, cells were allowed to migrate towards 10% lipid stripped FBS (chemoattractant) in the presence or absence of S1P (100 nM) for 16 h. The non-migrated cells on the top of migration insert were removed with a cotton swab. The migrated cells on the insert membrane bottom were fixed with 2% paraformaldehyde for 10 min, followed by staining with 0.1% crystal violet (20% methanol) for 5 min. Next, the membranes were washed with PBS and water and allowed to dry for overnight. The migrated stained cells were counted using 40× magnification from eight microscopic fields per insert.

### 2.9. Measurement of Intracellular [Ca^2+^]_i_

Cells grown on poly-lysine coated 25 mm round coverslips were loaded with 2 μM Fura-2-AM in HBM (HEPES buffered medium (in mM): 118 NaCl, 4.6 KCl, 1 CaCl_2_, 10 glucose and 20 HEPES. The pH was adjusted to 7.4 with Tris base) for 18 min at 37 °C and then transferred to a perfusion chamber. Single cell imaging data were acquired by using InCytIm2 fluorescence imaging system (Intracellular Imaging, Cincinnati, OH, USA). All experiments were performed at 37 °C under normal atmospheric conditions (21% O_2_, 0.038% CO_2_ in air). The cells were perfused in HBM at 37 °C and excited by alternating wavelengths of 340 and 380 nm using narrow band excitation filters. Fluorescence was measured through a 430 nm dichroic mirror and a 510 nm barrier filter with a Cohu CCD camera (20× objective used). One rationed image was acquired per second and on-line ratio values were converted to [Ca^2+^]_i_ by using a calibration curve. The data collected from 50–100 single cells per experiment was analyzed with the InCyt 4.5 software (Cincinnati, OH, USA) and further processed with Origin 6.0 software (Origin Lab Corporation, Northampton, MA, USA).

### 2.10. TRβ1 and Runx2 Staining of Patient Samples

Human Protein Atlas (HPA) is an up-to-date resource of the human tissue proteome including immunohistochemistry data [25]. The human tissue sample images data for TRβ1 and Runx2 expression were downloaded from HPA (www.proteinatlas.org, accessed on 12 January 2022) under the licence (v21.proteinatlas.org/about/licence, accessed on 12 January 2022). The normal thyroid, follicular thyroid carcinoma, and papillary thyroid carcinoma samples were from both female and male patients. The final images were acquired at 20× and 63× magnification using the case viewer software 2.3 (HISTECH Ltd., Budapest, Hungary).

### 2.11. High Throughput Data Mining and Gene Expression Analyses

The clinical and transcriptomic data of the Thyroid Carcinoma (THCA) cohort were downloaded from the GDC database (https://portal.gdc.cancer.gov/projects/TCGA-THCA, accessed on 10 March 2022). We included 502 thyroid tumors and 58 adjacent normal samples for transcriptome analysis. Among those tumor samples, 353 were papillary thyroid tumors, while 106 were follicular thyroid tumors. To assess gene expression levels in normal thyroid samples, we retrieved gene expression data (as raw count values) of 653 thyroid samples from the Genotype-Tissue Expression (GTEx) (https://gtexportal.org/home, accessed on 10 March 2022) for comparative analysis of gene expression. Only genes that express in at least 50% of the samples were assessed. DESeq2 was used for differential expression analysis between thyroid cancer groups and their matched para-cancerous samples [23,26]. A Benjamini–Hochberg adjusted *p* value of <0.05 was considered as statistical significance. The expression profiles of genes of interest (TRβ1 and Runx2) were also extracted from both retrieved datasets after performing variance stabilizing (VST) transformation and transcripts per million (TPM), followed by a log2 transformation for comparative analyses between cancerous tissues, cancer adjacent normal tissues, and controls.

### 2.12. Fast Gene Enrichment Analysis

The gene lists were pre-ranked and subsequently used as an input for fast gene set enrichment analysis (FGSEA) exactly as described in FGSEA packages [27]. Pathways of interest were investigated and further visualized by plotting enrichment score (ES) for each rank. An adjusted *p* value of 0.05 was used as a cut-off for significance.

### 2.13. Survival Analysis

Survival analysis was performed in R using survival 3.3-1 and survminer 0.4.9 packages as described elsewhere [28]. The patient population datasets were assessed as total (all thyroid patients), thyroid subtypes (papillary and follicular), or stages (Stages I, II, III and IV). The stages were categorized according to the eight edition of the American Joint Committee on Cancer [29]. For each gene (TRβ1 and Runx2), patients were divided into either high or low expression levels based on the median value. Survival curves were then estimated using the Kaplan-Meier method with the log-rank test for comparison. The *p* value less than 0.05 were considered as statistically significant.

### 2.14. Statistical Analysis

The results are presented as mean ± standard error of the mean (SEM) for at least three independent measurements. Benjamini–Hochberg method was used to correct the significances of differential expression datasets. Student’s t test was used when two means were compared. One-way ANOVA with Bonferroni’s post hoc tests were applied when three or more means were compared. Log-rank test was used for comparisons using Kaplan-Meiermethod. GraphPad Prism 9 program (GraphPad Software Inc., San Diego, CA, USA) was used for the statistical analyses. *p* values < 0.05 were considered statistically significant.

## 3. Results

### 3.1. Expression of TRβ1 and Runx2 in Normal and Thyroid Cancer Tissues

**Figure 1:**
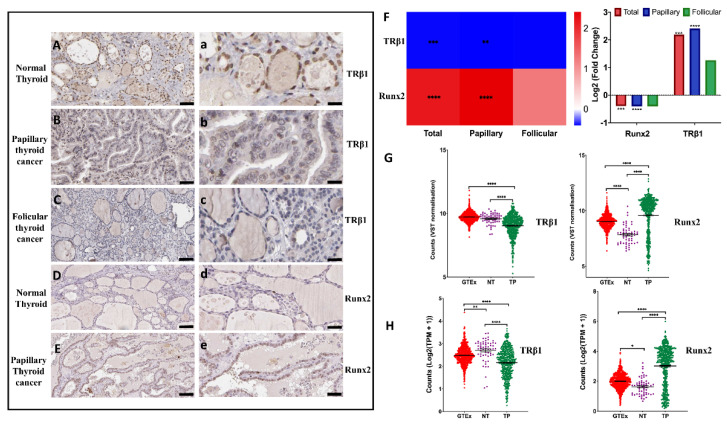
TRβ1 and Runx2 protein expression in human normal and thyroid cancer tissues. (**A**,**a**), Representative images of normal thyroid tissue; (**B**,**b**), Representative images of papillary thyroid cancer tissue; and (**C**,**c**), Representative images of follicular thyroid cancer tissue stained with TRβ1antibody CAB002009. (**A**–**C**), Scale bar 50 µm, 20× magnification, (**a**–**c**), scale bar 20 µm, 63× magnification. (**D**,**d**), Representative images of normal thyroid tissue and (**E**,**e**), Representative images of papillary thyroid cancer tissue stained with Runx2 antibody CAB068226. (**D**,**E**), Scale bar 50 µm, 20× magnification, (**d**,**e**), scale bar 20 µm, 63× magnification. The final images were acquired using case viewer software 2.3 (3DHITECH Ltd., Germany, https://www.3dhistech.com/research/software-downloads/, accessed on 12 January 2022). (**F**), Heatmap and bar plot to visualize differential expression of TRβ1 and Runx2 in tumor compared to their matched adjacent tissues in total, papillary and follicular thyroid cancer patients, respectively. The color intensity of heatmap represents log2 Fold Changes. In bar plot, data were presented as log2-transformed fold change calculated by DESeq2 algorithm. Significances have been corrected by the Benjamini–Hochberg method. (**G**,**H**), TRβ1 and Runx2 expression comparisons among normal controls (GTEx), thyroid para-cancerous tissues (NT) and their matched thyroid cancer tissues (TP). The expression profiles were extracted from the retrieved datasets (TCGA and GTEx) after variance stabilizing transformation (VST) or after converting raw counts to performing transcripts per million (TPM), followed by log2 transformation. Data were presented as mean ± SEM. One-way ANOVA test was used to assess statistical significance. **** *p* < 0.0001, *** *p* < 0.001, ** *p* < 0.01, * *p* < 0.05.

The Human Protein Atlas (www.proteinatlas.org, accessed on 12 January 2022) database contains each protein-coding gene expression using antibody-based profiling in normal and patient tissues. We downloaded the representative images of TRβ1 and Runx2 protein expression in normal and thyroid cancer patients’ tissues (Supplementary Figures S3 and S4). The images were modified, analyzed and are presented in (Figure 1Aa–Ee). We show that TRβ1 expression is downregulated in human papillary and follicular thyroid cancer, compared to the healthy normal thyroid tissues obtained from the patients (Figure 1Aa–Cc). In addition, we show that the expression of Runx2 is upregulated in papillary thyroid cancer patient tissues compared to healthy normal thyroid tissues of the patients (Figure 1Dd,Ee). These results are in line with a previous study that reported an upregulation of Runx2 expression in follicular thyroid cancer, compared to normal thyroid tissues [20].

### 3.2. Differential Expression of TRβ1 and Runx2 Genes in Normal Thyroid Tissues vs. Thyroid Cancer

Expression levels of TRβ1 and Runx2 genes were compared between thyroid tumor tissues (*n* = 502) and normal tumor-adjacent or para-cancerous thyroid tissues (*n* = 58) using RNA sequencing data from the TCGA Thyroid Carcinoma cohort. The expression of the TRβ1 gene was significantly down-regulated, while the Runx2 gene was significantly up-regulated in both the papillary and combined thyroid cancer tissues (Total), compared to respective tumor adjacent normal thyroid tissues (NT) (Figure 1F). Furthermore, in a comparison of TRβ1 and Runx2 expression in normal thyroid solid tissues, tumor adjacent normal thyroid tissues (NT) and thyroid tumor tissues (TP), we observed that the expression of TRβ1 was downregulated in thyroid tumor tissues (TP) compared to both normal thyroid tissues (GTEX) and tumor adjacent thyroid tissues (NT) (Figure 1G). However, Runx2 expression was upregulated in thyroid tumor tissues (TP) compared to both normal thyroid tissues (GTEX) and tumor adjacent thyroid tissues (NT) (Figure 1H).

### 3.3. MAPK Pathway Is Up-Regulated in Thyroid Tumors Compared with Adjacent Normal Thyroid Tissues

**Figure 2:**
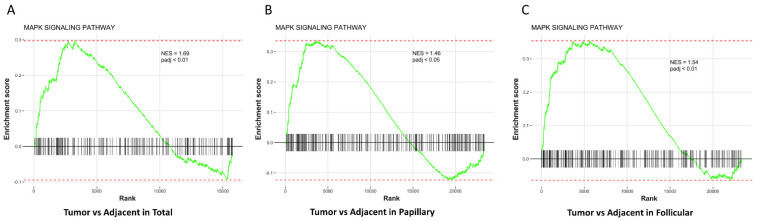
Gene set enrichment analysis (GSEA), enrichment plots based on normalized enrichment score (NES). (**A**). MAPK signaling pathway is significantly enriched in thyroid tumors (Total) vs. adjacent normal thyroid tissues (NES = 1.69, padj < 0.01). (**B**). MAPK signaling pathway is significantly enriched in papillary thyroid tumors vs. adjacent normal thyroid tissues (NES = 1.49, padj < 0.05). (**C**). MAPK signaling pathway is significantly enriched in follicular thyroid tumors vs. adjacent normal thyroid tissues (NES = 1.54, padj < 0.01).

Mitogen-activated protein kinase (MAPK) pathway modulates tumorigenesis, metastasis and survival of several tumor types, including thyroid, breast and colorectal cancers [30,31,32,33]. Most of the mutations of papillary, follicular and anaplastic thyroid cancer activate MAPK and PI3K pathways to contribute in the aggressive progression of thyroid cancer [30]. Thus, we performed Gene set enrichment analysis (GSEA) on TCGA downloaded thyroid cancer patients’ datasets to investigate the involvement of these pathways in thyroid cancer. Our results showed that the normalized enrichment scores (NES) for Kyoto Encyclopedia of Genes and Genomes (KEGG) MAPK signaling pathway were significantly increased in all thyroid tumors (Total, Papillary, and Follicular) compared with, or plotted against, respective tumor adjacent normal thyroid tissues (Figure 2A–C).

### 3.4. High or Low Expression of TRβ1 and Runx2 Has no Effect on Survival of Thyroid Cancer

The TRβ1 and Runx2 genes expression datasets (low and high) available for all thyroid cancer types and stages (I–IV) were downloaded from The Cancer Genome Atlas (TCGA), followed by the survival curve analyses based on Kaplan-Meier estimates to calculate the survival probability of the patients. The results showed that the low or high expression of TRβ1 and Runx2 has no significant effect on survival probability of thyroid cancer patients (Supplementary Figures S1 and S2).

### 3.5. Store-Operated Calcium Entry Modulates the Calcium Dependent Expression of TRβ1 and Runx2

**Figure 3:**
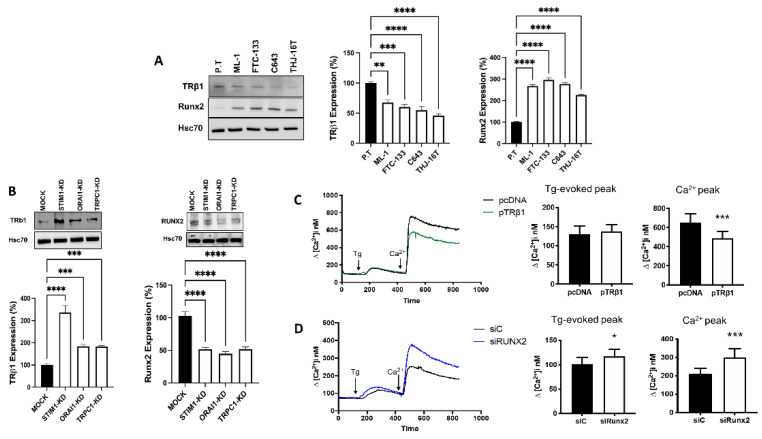
TRβ1 and Runx2 expression in normal and thyroid cancer cell lines and effects on store-operated calcium entry. (**A**), Expression of TRβ1 and Runx2 in thyroid cancer cell lines compared to normal human primary thyroid epithelial cells (P.T). (**B**), Protein expression of TRβ1 and Runx2 in shRNA expressing (MOCK), shRNA STIM1 expressing (STIM1-KD), shRNA ORAI1 expressing (ORAI1-KD) and shRNA TRPC1 expressing (TRPC1-KD) cells by Western blotting. Hsc70 was used as a loading control. The bar diagrams show the mean ± SEM (*n* = 3). ** *p* < 0.01, *** *p* < 0.001, **** *p* < 0.0001. (**C**), Representative trace showing changes in intracellular calcium when TRβ1 overexpressing ML-1 cells (pTRβ1) and control cells (pcDNA) in calcium-free buffer were treated with Thapsigargin (Tg, final conc. 1 µM) and when calcium was re-added (final conc. 1 mM). The left bar diagram shows the magnitude of the Tg-evoked peak in intracellular calcium, and the right bar diagram shows the magnitude of the changes in intracellular Ca^2+^ after calcium was re-added to the Tg-treated cells. The bars show the mean ± SEM of 35 cells. *** *p* < 0.001. (**D**), Representative trace showing changes in intracellular calcium in Runx2 silenced ML-1 cells (siRunx2) and control cells (siC) in calcium calcium-free buffer when treated with Thapsigargin (Tg, final conc. 1 mM) and when calcium was re-added (final conc. 1 mM). The left bar diagram shows the magnitude of the Tg-evoked peak in intracellular calcium, and the right bar diagram shows the magnitude of the changes in intracellular calcium after calcium was re-added to the Tg-treated cells. The bars show the mean ± SEM of 50 cells. * *p* < 0.05, *** *p* < 0.001.

We next investigated the expression of TRβ1 and Runx2 in normal primary thyroid cells, and in follicular and anaplastic thyroid cancer cells. In line with previous reports [13,14], we found that, TRβ1 expression is downregulated, while Runx2 is upregulated in follicular and anaplastic thyroid cancer cells, compared with primary thyroid epithelial cells (Figure 3A).

We have recently shown that downregulation of calcium signals e.g., by knocking down the transient receptor potential canonical channel 1 (TRPC1), or the stromal interacting molecule 1 (STIM1), dramatically decreases invasion and proliferation of thyroid cancer cells [23]. Here, we next investigated whether knockdown of STIM1, the plasma membrane calcium channel ORAI1, or the TRPC1 channel, affects the expression of TRβ1 and Runx2. We observed that knock down of all these factors potently upregulated the protein expression of TRβ1, whereas Runx2 was significantly downregulated (Figure 3B).

STIM1 has been shown to be involved in store-operated calcium entry (SOCE) and we have recently shown that knock down of STIM1 significantly attenuated SOCE in ML-1 cells [23]. As inhibiting SOCE modulated the expression of TRβ1 and Runx2, we therefore investigated if overexpression of TRβ1 or silencing Runx2 affects SOCE in ML-1 cells. Overexpression of TRβ1 did not affect the thapsigargin (Tg)-evoked calcium transient in ML-1 cells in a calcium-free buffer. However, when calcium was readded to the Tg-treated TRβ1 overexpressing ML-1 cells, there was a significant decrease in calcium entry, compared to control cells (Figure 3C). Surprisingly, silencing Runx2 with siRNA (siRunx2) significantly increased both the Tg-evoked calcium transient and calcium entry after calcium readdition to the Tg-treated cells, compared to control cells (Figure 3D). Furthermore, we investigated the SOCE in primary thyroid cells, and compared it to that observed in ML-1 cells. However, no significant difference was detected in either the Tg-evoked calcium transient in calcium-free buffer, or in the calcium entry when calcium was readded to the Tg-treated primary thyroid cells, compared to that observed in ML-1 cells.

### 3.6. TRβ1 Overexpression and Knockdown of Runx2 Attenuate Proliferation of ML-1 Cells

**Figure 4:**
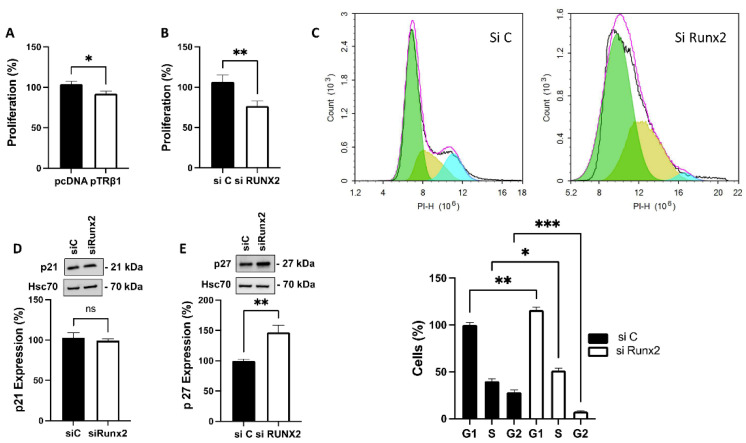
TRβ1 and Runx2 regulate the proliferation of thyroid cancer ML-1 cells. (**A**), Overexpression of TRβ1 (pTRβ1) decreased the proliferation of ML-1-cells after 48 h. The bar diagram shows the mean ± SEM (*n* = 3). * *p* < 0.05. (**B**), Silencing Runx2 (siRunx2) decreased the proliferation of ML-1 cells after 48 h. The bar diagram shows the mean ± SEM (*n* = 3). ** *p* < 0.01. (**C**), Runx2 knockdown decreased the proliferation of ML-1 cells by prolonging G_1_, S phases and a decrease in G_2_ phase of cell cycle. The bar diagram shows the mean ± SEM (*n* = 3). * *p* < 0.05, ** *p* < 0.01, *** *p* < 0.001. (**D**), Runx2 knockdown has no effect on p21 protein expression. A representative Western blot is shown. Hsc70 was used as a loading control. The bar diagram shows the mean ± SEM (*n* = 3). ns = not significant. (**E**), Runx2 knockdown increased p27 protein expression. A representative Western blot is shown. Hsc70 was used as a loading control. The bar diagram shows the mean ± SEM (*n* = 3). ** *p* < 0.01.

As knockdown of STIM1, ORAI1 or TRPC1 has been shown to attenuate both proliferation and invasion in ML-1 cells [22,23], we further investigated if overexpressing TRβ1, or knockdown of Runx2, has an effect on proliferation and invasion of ML-1 cells. For this purpose, the ML-1 cells were transfected with empty vector plasmid (pcDNA3) as control, TRβ1 plasmid (pTRβ1), non-targeting siRNA (si C) or si Runx2. We found that overexpression of TRβ1 (to a lesser extent), or knockdown of Runx2, significantly decreased the proliferation of ML-1 cells (Figure 4A,B). Since knockdown of Runx2 has such a clear effect on proliferation, we continued to investigate the mechanism. FACS analysis was used to study the cell cycle. In Runx2 knockdown ML-1 cells, there was a significant increase of the cell population in the G_1_ and S phases of cell cycle. In addition, the cell population was significantly decreased in the G_2_ phase (Figure 4C). We also investigated the protein expression of p21 and p27, the key regulators of the cell cycle. Runx2 did not affect the expression of p21, however, the expression of p27 was significantly increased (Figure 4D,E).

### 3.7. TRβ1 Overexpression or Knockdown of Runx2 Abolish S1P Evoked Invasion of ML-1 Cells

**Figure 5:**
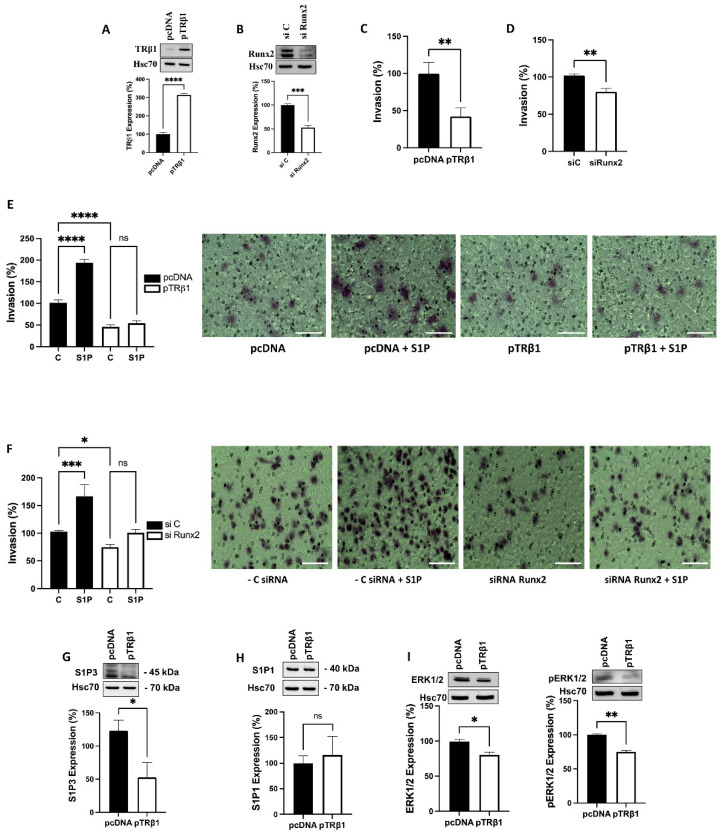
TRβ1 and Runx2 regulate basal and S1P-evoked invasion of thyroid cancer ML-1-cells. (**A**), A representative western blot showing the transfection efficacy of TRβ1 plasmid. Hsc70 was used as a loading control. The bar diagram shows the mean ± SEM (*n* = 3). **** *p* < 0.0001. (**B**), A representative western blot showing the transfection efficacy of siRunx2. Hsc70 was used as a loading control. The bar diagram shows the mean ± SEM (*n* = 3). *** *p* < 0.001. (**C**), Overexpression of TRβ1 (pTRβ1) decreased the invasion of ML-1 cells compared to control transfected cells (pcDNA). The bar diagram shows the mean ± SEM (*n* = 3). ** *p* < 0.01. (**D**), Runx2 knockdown decreased the invasion of ML-1 cells. The bar diagram shows the mean ± SEM (*n* = 3). ** *p* < 0.01. (**E**), Overexpression of TRβ1 attenuated basal and abolished S1P-evoked invasion in ML-1 cells. The bar diagram shows the mean ± SEM (*n* = 3). ns = not significant, **** *p* < 0.0001. The representative images of invasion inserts are shown. The scale bar is 100 µm. (**F**), Runx2 knockdown attenuated basal and S1P-evoked invasion in ML-1 cells. The bar diagram shows the mean ± SEM (*n* = 3). ns = not significant, * *p* < 0.05, *** *p* < 0.001. The representative images of invasion inserts are shown. The scale bar is 100 µm. (**G**), Overexpression of TRβ1 decreased the protein expression of S1P3 in ML-1 cells. A representative Western blot is shown. Hsc70 was used as a loading control. The bar diagram shows the mean ± SEM (*n* = 3). * *p* < 0.05. (**H**), Overexpression of TRβ1 had no effect on the protein expression of S1P1 in ML-1 cells. A representative Western blot is shown. Hsc70 was used as a loading control. The bar diagram shows the mean ± SEM (*n* = 3). ns = not significant. (**I**), Representative western blots showing the expression of ERK1/2 and pERK1/in TRβ1 overexpressing cells Hsc70 was used as a loading control. The bar diagram shows the mean ± SEM (*n* = 3). * *p* < 0.05, ** *p* < 0.01.

Sphingosine-1 phosphate (S1P), a bioactive lipid, potently evokes invasion of thyroid cancer ML-1 cells. This is mediated by the pro-migratory S1P receptors 1 and 3 [24,34]. Thus, we investigated the effects of overexpression of TRβ1, or knockdown of Runx2, on basal and S1P-evoked invasion in ML-1 cells. In control experiments we show that, transfecting the cells with a TRβ1 plasmid significantly restored TRβ1 expressional levels, while transfecting with a Runx2 siRNA potently downregulated Runx2 in the cells (Figure 5A,B). We found that both overexpression of TRβ1, or knockdown of Runx2, attenuated not only the basal invasion, but also abolished the S1P-evoked increase in the invasion of ML-1 cells (Figure 5C–F). In addition, we show that this abolished effect of S1P in TRβ1 overexpressing ML-1 cells was due, at least in part, to the downregulation of the pro- migratory S1P receptor 3, but not S1P receptor 1 (Figure 5G,H). No effect was observed on the anti-migratory S1P2 receptor expression. However, in Runx2 knockdown cells, we did not get a significant effect on the expression of the pro-migratory receptors S1P1 and S1P3. Furthermore, we observed a significant decrease in total ERK1/2 and pERK1/2 expression in pTRβ1 expressing ML-1 cells, compared to pcDNA control cells (Figure 5I).

### 3.8. Overexpression of TRβ1, but Not Runx2, Restored Thyroid Specific Protein Expression

**Figure 6:**
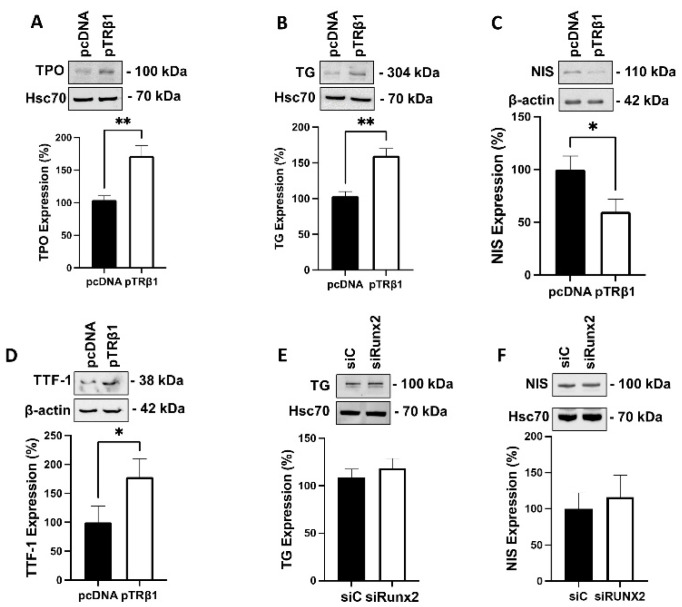
TRβ1 overexpression modulates thyroid specific proteins expression in ML-1 cells. (**A**), The protein expression of thyroperoxidase (TPO) was increased in TRβ1 overexpressing ML-1 cells (pTRβ1), compared to control cells (pcDNA). A representative Western blot is shown. Hsc70 was used as a loading control. The bar diagram shows the mean ± SEM (*n* = 3). ** *p* < 0.01. (**B**), The protein expression of thyroglobulin (TG) was increased in TRβ1 overexpressing ML-1 cells compared to control cells. A representative Western blot is shown. Hsc70 was used as a loading control. The bar diagram shows the mean ± SEM (*n* = 3). ** *p* < 0.01. (**C**), The protein expression of sodium/iodide symporter (NIS) was decreased in TRβ1 overexpressing ML-1 cells compared to control cells. A representative Western blot is shown. b-actin was used as a loading control. The bar diagram shows the mean ± SEM (*n* = 3). * *p* < 0.05. (**D**), The protein expression of thyroid transcription factor 1 (TTF-1) was increased in TRβ1 overexpressing ML-1 cells compared to control cells. A representative Western blot is shown. b-actin was used as a loading control. The bar diagram shows the mean ± SEM (*n* = 3). * *p* < 0.05. (**E**), Silencing Runx2 (siRunx2) had no effect on the protein expression of TG in ML-1 cells. A representative Western blot is shown. Hsc70 was used as a loading control. The bar diagram shows the mean ± SEM (*n* = 3). (**F**) Silencing Runx2 had no effect on the protein expression of NIS in ML-1 cells. A representative Western blot is shown. Hsc70 was used as a loading control. The bar diagram shows the mean ± SEM (*n* = 3).

Thyroid specific proteins, such as thyroperoxidase (TPO), thyroglobulin (TG), and sodium/iodide symporter (NIS), have been shown to be downregulated in thyroid cancer [35]. In addition, we have recently shown that knock down of STIM1 increased the expression of these proteins [23]. Therefore, we investigated whether overexpression of TRβ1, or Runx2 knockdown, affects the protein expression of TPO, TG, and NIS in ML-1 cells. As shown in Figure 6A–C, overexpression of TRβ1 increased the expression of TPO and TG, but surprisingly, decreased the expression of NIS. Furthermore, we investigated if overexpression of TRβ1 affects the protein expression of thyroid transcription factor 1 (TTF-1), which regulates the expression of several thyroid specific proteins, such as TPO, TG and NIS. The expression of TTF-1 has also been shown to be decreased in undifferentiated forms of thyroid cancer [36]. As shown in Figure 6D, the expression TTF-1 was upregulated in TRβ1 overexpressing ML-1 cells, in comparison with control cells. We did not observe any significant effect of Runx2 knockdown on expression of these thyroid-specific proteins in ML-1 cells (Figure 6E,F).

## 4. Discussion

We show here that the expression of the nuclear transcription factors TRβ1 and Runx2 is regulated by calcium in thyroid cancer cells. The knockdown of the calcium signalling proteins TRPC1, STIM1 and ORAI, increased the expression of TRβ1, but decreased that of Runx2 in human follicular thyroid cancer ML-1 cells. Previously, Carr et al. have shown that the expression of TRβ1 is downregulated, whereas Runx2 is upregulated, in thyroid cancer cell lines, compared to SV40 genome transduced immortalized normal primary thyroid follicular epithelial (Nthy-ori 3-1) cells [13]. However, some concerns have been raised as to the precise identity of some of the cell lines [37,38]. We observed that the expression of TRβ1 is downregulated, and that of Runx2 is upregulated, in verified human thyroid cancer cell lines, patient tissue samples and in clinical transcriptomic data, compared to the corresponding controls, i.e., human primary thyroid epithelial cells, tumor adjacent normal tissues and normal healthy patient tissues, respectively. These results are in line with the expressional patterns of TRβ1 and Runx2 as observed in lung cancer and breast cancer [39,40].

The importance of both TRβ1 and Runx2 has been investigated in several forms of cancer, including breast and thyroid cancer. In these cancers, TRβ1 has been considered to function as a tumor suppressor, and Runx2 as an oncogene [39,41]. Several investigations have shown that overexpression of TRβ1 reduces tumor growth, invasion, and metastasis in thyroid cancer [42,43]. Our results show that, overexpressing TRβ1 significantly reduced the invasion, and to a lesser extent proliferation, of ML-1 cells. These results are in line with earlier investigation [44]. Similarly, silencing Runx2 has been shown to reduce the invasion of WRO thyroid cancer cells, but no effect on migration was observed [20]. We showed that silencing Runx2 in ML-1 cells significantly attenuated both proliferation and invasion. As the decrease in proliferation in these cells was rather pronounced, we further investigated this. The decrease in proliferation is probably, in part, due to the increased expression of p27 that we observed. p27 is an inhibitor of cyclin dependent kinase (cdk), and inhibits the formation of cycline D1/cdk complexes during the G_0_ and G_1_ phase of the cell cycle. The expression of p27 is usually reduced in poorly differentiated thyroid cancer [45]. Thus, the increased expression of p27 probably explains, in part, the prolonged G1 phase of the cell cycle. In addition, downregulation of Runx2 also prolonged the S phase and shortened the G_2_ phase of the cell cycle.

Sphingolipids, in particular S1P, have been shown to be potent inducers of cancer invasion and metastasis [46]. We have previously shown that S1P potently stimulates migration and invasion in follicular thyroid cancer cells [24,34]. Our present investigation showed that, overexpression of TRβ1, or silencing of Runx2, decreased both the basal and the S1P-evoked invasion of ML-1 cells. Furthermore, overexpression of TRβ1 also significantly decreased the expression of S1P3. Both S1P1 and S1P3 have been shown to mediate the pro-migratory function of S1P in ML-1 cells [24,34]. Furthermore, the phosphorylation of ERK1/2, an important kinase for the S1P-evoked invasion of ML-1 cells [34], was significantly decreased. Since increased proliferation and invasion are essential hallmarks of cancer [47], our results thus suggest that by overexpressing TRβ1, or silencing Runx2 in ML-1 cells, their aggressive characteristics change towards a more less aggressive, normal phenotype.

The calcium signals created by SOCE play many critical roles in the promotion of tumor growth, migration, and invasion in many different cancers [48,49]. STIM1 is involved in SOCE, and we have recently shown that knocking down STIM1 attenuates SOCE and invasion of ML-1 cells [23]. As knocking down calcium entry increased TRβ1 and decreased Runx2 expression, we were interested in investigating whether overexpressing TRβ1, or silencing Runx2, regulate calcium entry in ML-1 cells. Overexpression of TRβ1 significantly attenuated calcium entry in ML-1 cells. Surprisingly, silencing Runx2 had the opposite effect, and significantly increased calcium entry in ML-1 cells. As SOCE is mediated by STIM1 and ORAI1 [50,51], we investigated if overexpression of TRβ1, or silencing Runx2, affected the expression of these two proteins. Interestingly, the protein expression of STIM1 or ORAI1 was not affected by overexpression of TRβ1, or by silencing of Runx2, in ML-1 cells. Therefore, the mechanism by which TRβ1 and Runx2 regulate calcium entry in ML-1 cells is still unknown, and thus remains to be investigated.

We observed that overexpression of TRβ1 increased the expression of thyroid specific proteins such as TPO and TG, but also the expression of TTF-1, which in turn, has been shown to regulate the expression of several thyroid specific proteins [36]. As thyroid specific proteins, such as TPO, TG and NIS, have been shown to be downregulated in poorly differentiated forms of thyroid cancer [35], our results suggest that by overexpressing TRβ1, ML-1 cells start to re-differentiate from a poorly differentiated phenotype towards a more normal thyroid phenotype. Interestingly, overexpression of TRβ1 decreased the expression of NIS in ML-1 cells. This is a surprising observation since the other thyroid specific proteins were clearly upregulated.

## 5. Conclusions

Taken together, our results suggest that the expression of TRβ1 and Runx2 is calcium-dependent. This likely is of functional significance in the context of some malignancies, including thyroid cancer. In line with this, we observed that overexpression of TRβ1 led to changes in ML-1 thyroid cancer cells from an aggressive, and poorly differentiated phenotype, towards a more normal thyroid phenotype. In view of this, the expression of TRβ1 in cancer cells could be a promising therapeutic approach to consider in design of better and more effective treatments of some thyroid cancers. Future investigations, including the use of thyroid-specific nanoparticles [52], could thereby be of particular interest.

## Figures and Tables

**Table 1:** shRNA-lentiviral vectors, primers and siRNA information.

Product	Gene ID	Nucleotide Sequence (5′-3′)
**shRNA**	Tcf25, TRCN0000190494, 66855 STIM1, TRCN0000149588, 6786 ORAI1, TRCN0000161221, 84876 TRPC1, TRCN0000043998, 7220	CCGGGCCTCTGTCTCCCAAATGTTACTCGAGTAACATTTGGGAGACAGAGGCTTTTTTG CCGGCGATGAGATCAACCTTGCTAACTCGAGTTAGCAAGGTTGATCTCATCGTTTTTTG CCGGGAAACTGTCCTCTAAGAGAATCTCGAGATTCTCTTAGAGGACAGTTTCTTTTTTG CCGGGCCCACCTGTAAGAAGATAATCTCGAGATTATCTTCTTACAGGTGGGCTTTTTG
**Primers**	TRβ1 forward Tm (°C) 57.3 TRβ1 Reverse Tm (°C) 57.3	TCTTCCCCCCTTTGTTCTTG ACTACTTCCCTTTTCCCTCC
**siRNA**	Non-targetting control siRNA siRNA TRβ1 siRNA Runx2	CCU ACA UCC CGA UCG AUG AUG AUC AUC ACA CCA GCA AUU A GCU ACC UAU CAC AGA GCA A

## Data Availability

Image credits: Human Protein Atlas (HPA), the immunohistochemistry (IHC) images downloaded for this study are available at following URLs (acsessed on 12 January 2022): v21.proteinatlas.org/ENSG00000124813-RUNX2/tissue/thyroid+gland; v21.proteinatlas.org/ENSG00000124813-RUNX2/pathology/thyroid+cancer; v21.proteinatlas.org/ENSG00000151090-THRB/tissue/thyroid+gland; v21.proteinatlas.org/ENSG00000151090-THRB/pathology/thyroid+cancer.

## Supplementary Materials

The following supporting information can be downloaded at: https://www.mdpi.com/article/10.3390/cancers14235838/s1, Figure S1: Kaplan-Meier survival analysis for low and high expression of TRβ1 in (**A**), thyroid cancer (Total), (**B**), papillary, (**C**), follicular and (**D**–**G**), stages of thyroid cancers (I–IV), based on TCGA data analysis.; Figure S2: Kaplan-Meier survival analysis for low and high expression of Runx2 in (**A**), thyroid cancer (Total), (**B**), papillary, (**C**), follicular and (**D**–**G**), stages of thyroid cancers (I–IV), based on TCGA data analysis. Figure S3: THRB1 Tissue Expression in normal thyroid tissue vs. Papillary and Follicular thyroid cancer tissues. Figure S4: Runx2 Tissue Expression in normal thyroid tissue vs. Papillary thyroid cancer tissues.
