# Supplementary material for: Suppression of Calcium Entry Modulates the Expression of TRβ1 and Runx2 in Thyroid Cancer Cells, Two Transcription Factors That Regulate Invasion, Proliferation and Thyroid-Specific Protein Levels
Source: Cancers (Basel). 2022 Nov 26;14(23):5838. doi: 10.3390/cancers14235838 (PMC9740761; doi:10.3390/cancers14235838)
Supplement: Supplementary file 1 [file cancers-14-05838-s001.zip › cancers-1945152-supplementary.pdf]

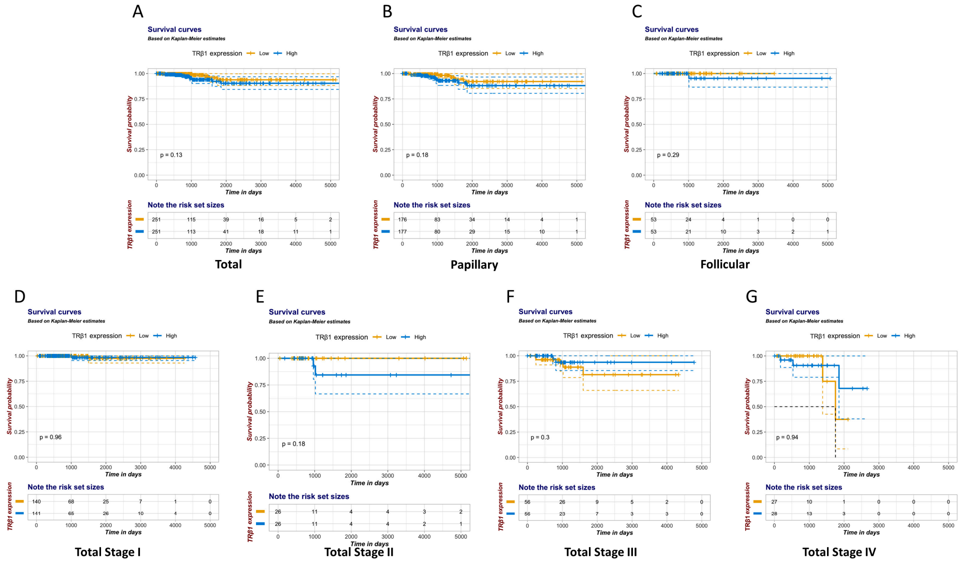

Figure S1. Kaplan-Meier survival analysis for low and high expression of TRβ1 in (**A**)**,** thyroid cancer (Total), (**B**)**,** papillary, (**C**), follicular and (**D-G**)**,** stages of thyroid cancers (I – IV), based on TCGA data analysis.

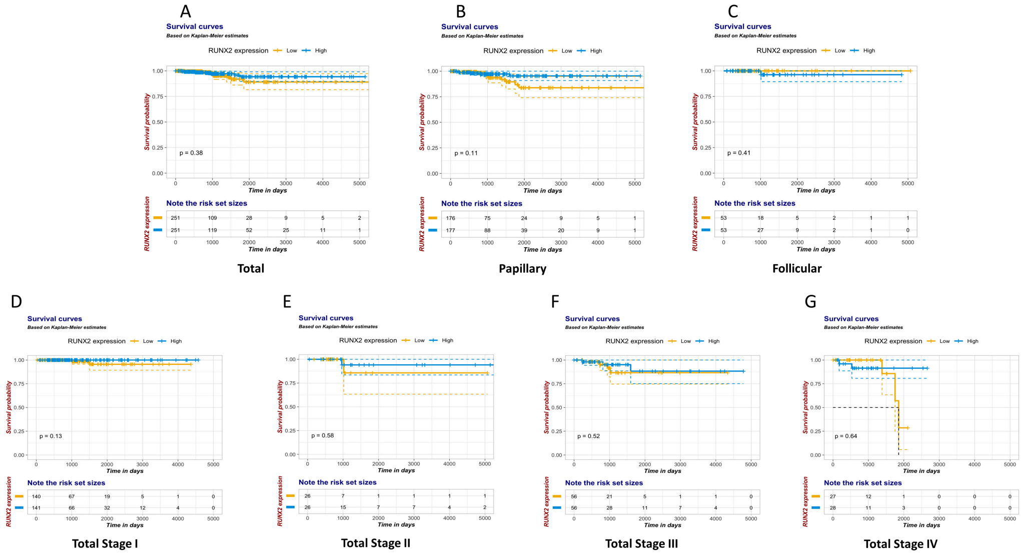

Figure S2. Kaplan-Meier survival analysis for low and high expression of Runx2 in (**A**)**,** thyroid cancer (Total), (**B**)**,** papillary, (**C**)**,** follicular and (**D-G**), stages of thyroid cancers (I – IV), based on TCGA data analysis.

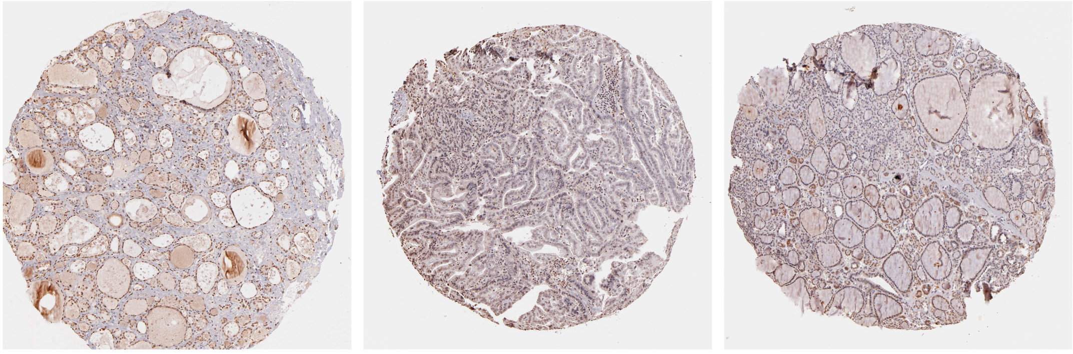

CAB002009_1501_**Normal** tissue, NOS_M-00100_T-96000_1

CAB002009_985_**Papillary** adenocarcinoma, NOS_M-82603_T-96000_7

CAB002009_923_**Follicular** adenoma carcinoma, NOS_M-83303_T-96000_6

Figure S3. THRB1 Tissue Expression in normal thyroid tissue vs Papillary and Follicular thyroid cancer tissues.

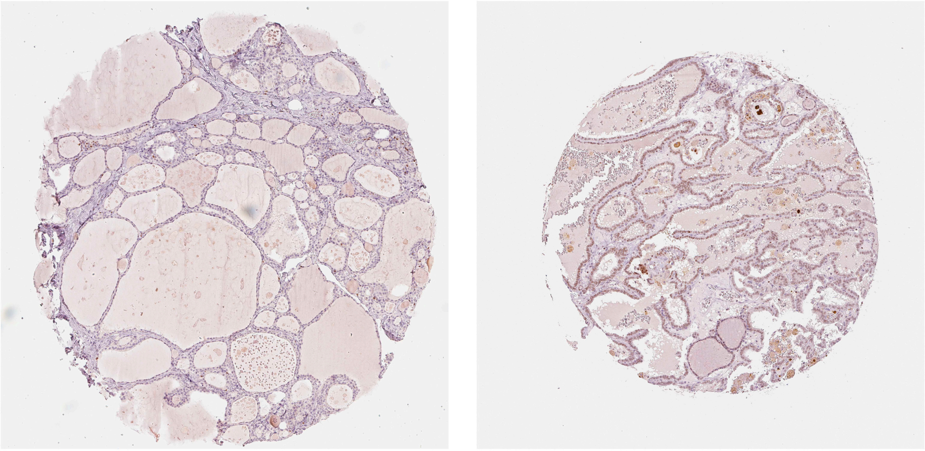

CAB068226_1672_**Normal tissue**, NOS_M-00100_T-96000_1

CAB068226_189_**Papillary** adenocarcinoma, NOS_M-82603_T-96000_3

Figure S4. Runx2 Tissue Expression in normal thyroid tissue vs Papillary thyroid cancer tissues.
